# Commentary: Evaluation of ultrasound accuracy in thyroid mass measurement and its impact on 131I treatment for Graves’ disease

**DOI:** 10.3389/fendo.2025.1675733

**Published:** 2025-09-30

**Authors:** Yuxue Qi, Jiandi Zheng

**Affiliations:** ^1^ The Second Clinical Medical College of Zhejiang Chinese Medical University, Hangzhou, Zhejiang, China; ^2^ Department of Endocrine and Metabolic Diseases, Hangzhou Red Cross Hospital, Hangzhou, Zhejiang, China

**Keywords:** thyroid mass, Graves’ disease, 131I treatment efficacy, clinical predictive model, CT calibration

Dear Editor,

I read with great interest the recent article by Li et al. evaluating ultrasound accuracy in thyroid mass measurement and its impact on radioiodine therapy for Graves’ disease (1). This study used CT as the gold standard for the first time, and systematically revealed the problem of significant and systematic underestimation of ultrasound in the evaluation of large thyroid masses >20g. Through the comparison of US and CT image data of 192 patients, it was quantitatively confirmed that the average deviation of US measurement was 16.65g and the consistency was extremely poor. Based on the treatment data of 1584 Graves’ disease patients, it was determined that thyroid mass was an independent key factor affecting the success rate of the first ¹³¹I treatment, and 35.6g was determined as an important critical value. This study directly challenges the routine clinical practice of relying on US to calculate ¹³¹I dose, and proposes that for thyroid gland >20g, CT calibration is recommended before treatment to improve the dose accuracy, which provides an important evidence-based basis for optimizing the individualized treatment of Graves’ disease.

While these findings provide substantial clinical value, a few aspects could be further strengthened to enhance the study’s impact.

The main deficiency of this paper is that the clinical practical value of the prediction model established is still limited. Although several important factors such as disease duration, FT4, RAIU, ¹³¹I dose, and thyroid mass were included, the predictive power (AUC = 0.663) of the model was only moderate, and the sensitivity was also low, which limited the direct application of the model in individualized accurate prediction. In addition, higher ¹³¹I doses were observed to be associated with an increased risk of treatment failure, which was contrary to conventional wisdom and some studies. The authors attributed this finding only to the lower upper limit of ¹³¹I dose used in their center, without providing further explanation, leaving this key result underinterpreted (2). Finally, the follow-up of only 6 months is relatively short to assess the effect of the CT-calibration strategy on long-term outcomes and to capture possible late efficacy responses.

Despite these opportunities for refinement, this work meaningfully advances our approach to Graves’ disease. It convincingly argues that precision in thyroid volumetry is not merely technical—it is therapeutic. I look forward to seeing these insights integrated into future guidelines.
